# Effects and Interactions of Medium Components on Laccase from a Marine-Derived Fungus Using Response Surface Methodology

**DOI:** 10.3390/md7040672

**Published:** 2009-11-25

**Authors:** Donna D’Souza-Ticlo, Sandeep Garg, Chandralata Raghukumar

**Affiliations:** 1 Marine Biotechnology Laboratory, National Institute of Oceanography, Council of Scientific & Industrial Research, Dona Paula, Goa 403 004, India; 2 Department of Microbiology, Goa University, Taleigao Plateau, Goa 403 206, India

**Keywords:** marine-derived basidiomycete, laccase, response surface methodology, Central Composite Design and Plackett Burman Design

## Abstract

The effects of various synthetic medium components and their interactions with each other ultimately impact laccase production in fungi. This was studied using a laccase-hyper-producing marine-derived basidiomycete, *Cerrena unicolor* MTCC 5159. Inducible laccases were produced in the idiophase only after addition of an inducer such as CuSO_4_. Concentration of carbon and nitrogen acted antagonistically with respect to laccase production. A combination of low nitrogen and high carbon concentration favored both biomass and laccase production. The most favorable combination resulted in 917 U L^−1^ of laccase. After sufficient growth had occurred, addition of a surfactant such as Tween 80 positively impacted biomass and increased the laccase activity to around 1,300 U L^−1^. Increasing the surface to volume ratio of the culture vessel further increased its activity to almost 2,000 U L^−1^.

## 1. Introduction

Laccases (benzenediol: oxygen oxidoreductase, EC 1.10.3.2) are multi-copper containing enzymes which reduce molecular oxygen to water and simultaneously perform one electron oxidation of various aromatic substrates [1]. This broad substrate specificity of laccase has resulted in a large number of biotechnological applications. These include the decolorization and detoxification of textile dyes and effluents, pulp delignification, removal of phenolics from wines and other beverages, transformation of antibiotics, steroids and many aromatic compounds [2].

Laccases from marine sources with thermo- and halo-tolerance have potential in bioremediation of wastewaters with high pH and salt content. A marine-derived basidiomycete *Cerrena unicolor* MTCC 5159 isolated from decaying mangrove wood was found to produce a large number of laccase isoforms of which, at least one of them was thermostable, halotolerant and heavily glycosylated [3]. These laccases decolorized several dyes and effluents [4]. Considering the bioremediation potential of these laccases, we have attempted to study the medium components that affect their production and whether these affect laccase production in the marine-derived fungus differently when grown in a distilled water medium.

Classical methods that use sequential manipulation of a single parameter do not take into consideration the interactions between different factors. Moreover, they are work and time exhaustive. Response surface methodology has eliminated these drawbacks. It can also be used to evaluate the relative significance of several variables simultaneously [5]. The aim of this study was to apply the Plackett Burman Design (PBD), to determine which medium components had the most significant effect on laccase titer and the Central-Composite Design (CCD) to determine the interactions of these significant components. Some major variables affecting laccase and biomass production were separately investigated.

## 2. Results and Discussion

Laccases and their various applications in degradation of xenobiotics by aquatic [6,7], obligate and marine-derived fungi [8,9] have been reported. Several such fungi are reported to produce novel secondary metabolites and enzymes which have not been reported from their terrestrial counterparts [10]. The present isolate of *Cerrena unicolor* MTCC 5159 is reported to produce halotolerant laccase [3] and degrade raw textile mill effluents [11]. Although it showed 99% identity to *C. unicolor* 18 S rDNA, it showed only 91% identity with ITS rDNA. However it showed 99% identity (ITS rDNA) with unidentified basidiomycetes associated with marine sponges suggesting its marine origin. This fungus appears to be a marine-adapted strain of the terrestrial *C. unicolor* as evidenced by its growth and laccase production and degradation of effluents in media containing seawater [4]. Also, one of its isozymes Lac IId, was not inhibited by NaCl up to 0.3 M, above which it was only reversibly inhibited [12]. It retained 75% of its activity in the presence of half strength seawater [3]. Further, isoelectric focusing of the partially purified culture supernatant of this isolate when grown in distilled water produced maximum number of laccase isozymes around pI 4 whereas when grown in medium containing full strength seawater, the pI was around 7 [3]. The optimum temperature for laccase in the crude culture filtrate was 60 °C whereas for purified Lac IId, it was 70 °C [3].

This marine-adapted isolate of *C.unicolor* differed from its terrestrial counterparts in the following ways: Laccase from a terrestrial strain of *C.unicolor* showed activity at 40 °C [13]. Lac IId of this marine-adapted had a half life of 90 min at 70 °C in contrast laccase from the terrestrial *C.unicolor* strain 137 Lacc I, lost its complete activity in less than 10 min at 70 °C and Lacc II had a half life of only 10 min at 70 °C [14].

**Figure 1.**
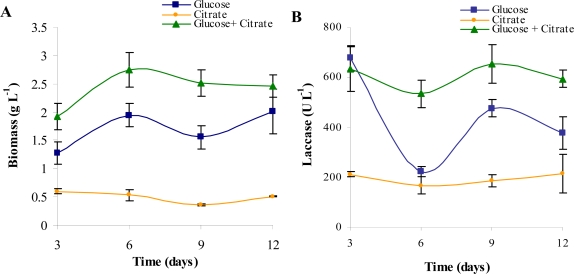
Effect of glucose and citrate on (a) Biomass and (b) Laccase production in a low nitrogen medium.

Laccases are produced in excess when the laccase-producing fungi are grown at their optimum pH which is 5 [1]. In most of the studies, pH levels are set between 4.5 and 6, prior to inoculation [15–17]. To estimate the effect of individual components accurately, the synthetic culture medium was prepared with distilled water. Citrate-phosphate buffer was used to maintain the pH at 4.5. Since citrate phosphate buffer too contains assimilable carbon, the interaction between the buffer and the designated carbon source was determined. It was found that both glucose (carbon source) and citrate (buffer component) serve as independent sources of carbon for both laccase and biomass production (Figures 1a and 1b). Higher amount of biomass build-up in the medium containing solely glucose probably was merely a function of its relatively much higher concentration rather than its assimilability. When citrate was used as the sole carbon source, a stable but low trend of laccase production was observed, whereas when only glucose was present, the laccase titer increased along with a large temporal variation. When both glucose and citrate were present together, the effect was additive on biomass (Figure 1a) and a decreased temporal variation in laccase production (Figure 1b) was observed. Thus, citrate-phosphate was used in the medium as a buffer and maintained at a constant basal level in all further experiments. However, the amount of carbon from citrate that is utilized by the fungus merits further investigation.

The screening of medium components for their influence was carried out using PBD. The coded and uncoded values for all of the eight experimental Trials used in the PBD are shown in Table 1

Table 2 presents the biomass and laccase values of the PBD sets determined at various sampling intervals, with and without an additional spike of CuSO_4_ (on day 6).

In majority of the cases in both the sets, spiked as well as unspiked, maximum biomass was observed on day 9 whereas maximum laccase production occurred on day 6.

Laccases are reported to be induced in the presence of copper ions [18]. Palmieri *et al*., 2000 [19] reported a fifty-fold increase in laccase activity in *Pleurotus ostreatus*, a white-rot basidiomycete after the addition of 0.15 mM CuSO_4_. However high levels of CuSO_4_ in the cultivation medium have been shown to have toxic effects on several white-rot fungi [18,20]. Preliminary studies on the effect of CuSO_4_ on biomass were carried out using concentrations of CuSO_4_ up to 2 mM with increments of 0.25 mM, added on the day of inoculation (data not shown). Here, 0.375 mM CuSO_4_ was found to induce laccase without affecting biomass buildup. Hence CuSO_4_ was added to the medium on the day of inoculation at either 0.375 or 0.0375 mM (according to the Trial set-up) yet, statistical analysis of these results indicated a delay in biomass accumulation.

Table 3 illustrates the impact that each medium component individually had on biomass production. Ammonium chloride and NaCl had a negative coefficient suggesting that higher concentrations of either component would drastically and negatively affect biomass accumulation. Glucose had a comparatively positive impact throughout whereas CuSO_4_ and Tween 80 had a positive impact from day 6 onwards. The level of impact also reflected how critical these components were, especially after 6 days of growth.

It was observed in the CuSO_4_ spiking experiment that the second addition of CuSO_4_ magnified the impact the medium components had on biomass production and the deviation between the spiked and unspiked set was large. After the additional spike of CuSO_4_, biomass production was not as good as the unspiked set.

Table 4 illustrates the impact that each medium component individually had on laccase production. Initially, all the medium components except NH_4_Cl, had a negative impact. However by day 12 the impact of glucose turned positive after which, although it fell drastically it did not turn negative. A delay in laccase production in the presence of high concentrations of glucose had been previously observed in *Trametes versicolor* [21]. Earlier it was suggested that easily assimilable components such as glucose, allow for constitutive laccase production but repress its induction in several fungi [22]. Both constitutive as well as inducible laccases are present in MTCC 5159, with the constitutive forms being produced at a lower level than the inducible ones [23]. An alternative to avoid this time delay in laccase production is to use a carbon source that is not very easily assimilable.

The impact of NH_4_Cl an inorganic nitrogen source except on day 3, was decidedly negative throughout. Its maximum negative impact coincided with maximum laccase production on day 6 (Table 4), indicating that the concentration of nitrogen was critical for laccase production. Ligninolytic systems of white-rot fungi are known to be activated during the secondary metabolic phase of the fungus and are often triggered by nitrogen depletion [24].

Sodium chloride had a stable negative impact on laccase production and on day 6, its negative impact increased six-fold coinciding with maximum laccase activity. This fungus has been previously shown to produce maximum laccase in seawater of 25 ppt salinity [4]. However, here the addition of sodium chloride had an inhibitory effect on laccase activity (Table 4). Apparently, seawater with its other constituents supports growth and laccase production in this fungal strain and sodium chloride may not be a good replacement for it. The maximum NaCl concentration used was 1%, whereas NaCl is known to reversibly inhibit laccase activity of this fungus only above 1.74% [12]. Hence laccase production and not its’ activity was adversely affected by sodium chloride. This adverse effect could also be due to the combined stress of the synthetic nature of the medium, as it is known that fungi have a preference for organic substrates [25].

After a very negative impact on day 3, the impact of CuSO_4_ turned positive with a very high magnitude. Tween 80 followed a similar trend as that of CuSO_4_ and its impact was the highest among all the components tested. Some components affect the metabolism or growth rate while others, trigger laccase production [26,27]. The promoter regions of laccase genes have been shown to contain various recognition sites that are specific for heavy metals which when bound to, induce laccase production [28]. It seems that at the concentrations used here, CuSO_4_ and Tween 80 affected both growth as well as laccase production.

In the present case, CuSO_4_ spiking (2^nd^ addition of CuSO_4_) lead to a dramatic decrease in laccase production as seen from the laccase titer values in Table 2. From Table 4, it was observed that NaCl had a negative impact on laccase titer and CuSO_4_ spiking changed its impact to positive. This reversal of impact of NaCl after addition of CuSO_4_ was probably due to the dramatically lowered laccase titer values. Higher amounts of glucose and NH_4_Cl resulted in delayed laccase production. This may be due to enhanced primary growth of the fungus resulting in delayed production of laccase which is normally produced in the idiophase.

Although Tween 80 initially affected laccase accumulation adversely, later it showed a very significant positive impact. This may be due to either of the following reasons or a combination thereof; (1) The surfactant property of Tween 80 which emulsifies the fungal membrane aiding in the release of cell membrane-associated laccases [29] as well as in the secretion of the normal extracellular laccases. (2) The decrease in amount of evaporation of moisture due to the presence of Tween 80, which would otherwise have led to the concentration of medium components during prolonged incubation periods and thus either increasing adverse interactions between components or the precipitation of critical components. Although Tween 80 has been also known to increase the activity of the already secreted enzyme [29]. This was not the case here, as in an independent experiment, addition of a constant amount of Tween 80 to the cell-free culture filtrate, did not increase the laccase activity.

The initial negative impact of CuSO_4_ on laccase production suggests that either CuSO_4_ had an initial toxic effect on biomass accumulation or merely that the major laccases produced in this medium are not constitutive but of the inducible type and thus the mandatory lag before laccase production. Strangely, a spike of CuSO_4_ on day 6 did not yield the expected spurt in laccase production considering that sufficient biomass had accumulated to offset any toxicity resulting from CuSO_4_ addition. In fact, laccase production had been drastically reduced (Table 4) in such a way that the effect of this additional CuSO_4_ overshadowed the effects of all other medium components. Thus, CuSO_4_ was effective in increasing the laccase activity but when its concentration was increased by the addition of 0.375 mM CuSO_4_ at a later time, it had an adverse effect. Thus CuSO_4_ positively affected laccase production however the upper limit of CuSO_4_ concentration requires careful fine tuning. We hypothesize that the initial addition of CuSO_4_ is welcomed by the fungus since it is used in biomass accumulation. Metals are required by fungi for their normal growth and laccase synthesis as copper is a cofactor of laccase, *i.e.*, one laccase molecule requires four copper atoms for it to be fully functional [1]. However, the second addition of copper adversely affected laccase production contrary to the assumption that sufficient biomass had accumulated to offset the copper toxicity. Whether this toxicity was a universal effect or not, cannot be ascertained since sufficient growth had already occurred but laccase production was specifically negatively affected. Whether the synthesis of laccase or merely its activity was affected while assaying, due to the inhibiting and prolonged presence of copper, requires further investigation. Thus, a single addition of copper was preferred. The addition of CuSO_4_ at the time of inoculation resulted in delayed laccase production. This was probably a function of toxicity on growth and not due to the fact that laccase is generally produced in the idiophase. Thus, a single of addition of CuSO_4_ after sufficient growth had occurred would be ideal. This would overcome the toxic effect of copper on biomass yet retain its positive impact on laccase production. Hence, for all further experiments, a single addition of CuSO_4_ at 0.375 mM on day 6 was carried out. Laccase and biomass production were recorded 6 days after that *i.e.*, on day 12 since maximum laccase production occurred 6 days after the addition of CuSO_4_ [4].

Glucose and NH_4_Cl as the carbon and nitrogen source respectively are essential for biomass accumulation yet both these components at higher concentrations negatively affected biomass and laccase production. The CCD was used to study the interaction of both these components at varying concentrations. The design which includes the coded and uncoded values as well as the experimental, predicted and residual values are given in Table 5. Since NaCl had a negative impact on both laccase and biomass production (Tables 3 and 4), it was maintained at a constant low level of 0.1% (Table 5).

These values were fitted in a second order polynomial equation. The values of regression coefficients were calculated and the fitted equations (using uncoded values) for predicting biomass (Y_B_) and laccase (Y_L_) production were:

(1) $${\text{Y}}_{\text{B}}\;=\;1.575\;+\;(0.5211*\text{A})\;+\;(-0.1586*\text{B})\;+\;(-0.05917*\text{A}*\text{A})\;+\;(0.1224*\text{B}*\text{B})\;+\;(-0.2988*\text{A}*\text{B})$$

(2) $${\text{Y}}_{\text{L}}\;=\;115\;+\;(276*\text{A})\;+\;(-364*\text{B})\;+\;(10*\text{A}*\text{A})\;+\;(1358*\text{B}*\text{B})\;+\;(-634*\text{A}*\text{B})$$

Where A is glucose and B is NH_4_Cl concentration. Irrespective of whether biomass or laccase was concerned, glucose and NH_4_Cl interacted antagonistically with each other. Further for biomass, the linear coefficient of glucose was strongly positive whereas its quadratic coefficient was slightly negative. On the other hand, the interaction of glucose with NH_4_Cl was negative. The quadratic coefficient of NH_4_Cl for biomass indicated that biomass accumulation was not very dependant on NH_4_Cl concentration whereas for laccase, it appeared that only low concentrations favored laccase production. It had been experimentally proven that laccase production is highly dependent on the conditions of cultivation of the fungus [30] and media supporting high biomass does not necessarily support high laccase yields [31]. This probably acts as a sort of check on uncontrolled laccase production which would be harmful for the fungus since active growth requires the presence of metabolites and enzymes other than laccase as well. This would not be possible if the entire cell machinery had been routed to laccase production. Also, excessive concentrations of glucose could not be used since the quadratic coefficient of glucose for biomass accumulation, although positive was of a low magnitude. Excessive concentrations of glucose as a carbon source in the cultivation of laccase-producing fungi had an inhibitory effect on laccase titer [20].

**Figure 2.**
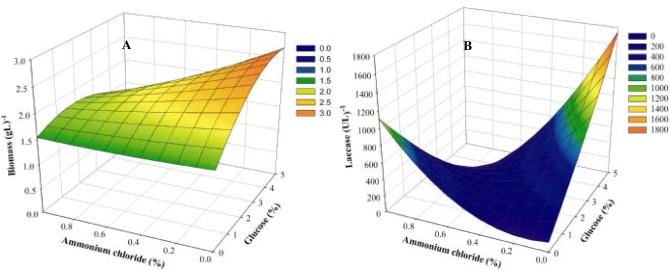
Surface plots of Biomass (a) and Laccase (b) production, illustrating the interaction between glucose (carbon) and ammonium chloride (nitrogen).

Surface plots for both biomass and laccase production elucidating the interaction between glucose and NH_4_Cl were plotted using the above equations (Figures 2a and 2b). Low concentrations of NH_4_Cl and high concentrations of glucose favored biomass production. The positive but low magnitude of the glucose coefficient indicated that biomass would increase to a limited extent with increasing glucose concentrations (Figure 2a). Levels of glucose and NH_4_Cl work antagonistically with each other, hence the inverted bell shaped curve obtained for laccase production (Figure 2b).

Thus a medium containing either a combination of high NH_4_Cl and low glucose concentration or a combination of low NH_4_Cl and high glucose concentration would be ideal for laccase production. Even though laccase activity is known to increase during the idiophase [32], for laccase production to occur, a critical amount of biomass is required. This is favored at high glucose concentrations. Thus, a high glucose and low NH_4_Cl concentration would yield sufficient biomass to produce laccase in an appreciable titer. However, the negative quadratic coefficient of glucose for biomass and the positive but low value for laccase, limits the maximum concentration of glucose that could be used. Beyond these concentrations both, biomass and laccase production would be detrimentally affected. The low nitrogen content of the synthetic culture medium mimics the nitrogen depletion that occurs when the fungus reaches its idiophase, triggering laccase production [24]. It is evident that the concentration of NH_4_Cl was more critical than glucose and this merits further investigation.

**Figure 3.**
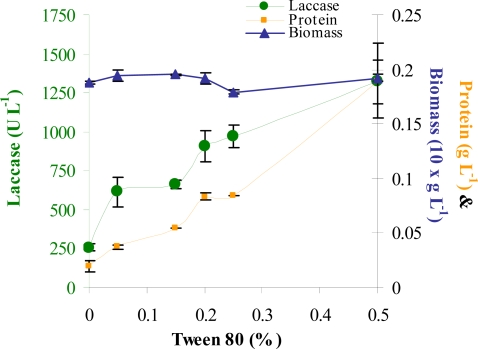
Effect of Tween 80 concentrations on laccase, biomass and protein production.

Tween 80 had been omitted from the experimental set up in CCD as its concentration was determined separately as seen in Figure 3. Increased Tween 80 concentrations did not significantly affect biomass production (Figure 3).

Both laccase and protein production showed significant dependence on Tween 80 concentration and both showed increased activity in the presence of higher concentrations. At lower concentrations, the majority of protein secreted was laccase. However beyond 0.25%, laccase specific activity decreased significantly even though there was still an increase in laccase titer. Also at higher Tween 80 concentrations, the medium became increasingly frothy due to its surfactant properties. Thus 0.25% Tween 80 was selected for further experiments.

**Figure 4.**
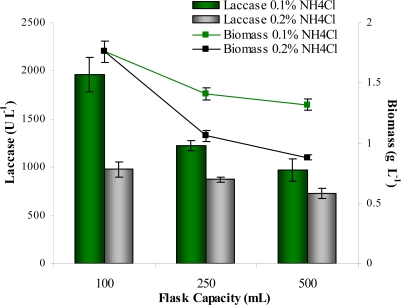
Effect of flask capacity on the production of biomass and laccase when grown in the presence of either 0.1 or 0.2% NH_4_Cl.

It is a well known fact that laccase is best produced under stationary conditions where the fungal mycelium is in maximum contact with the atmospheric surface [33]. The CCD results were validated using 3% glucose, 0.25% Tween 80 and NH_4_Cl at either 0.1 or 0.2% in varying flask capacities (Figure 4).

The surface to volume ratios of 100, 250 and 500 mL capacity flasks were 3.78, 2.64 and 1.93 respectively. At the concentrations of NH_4_Cl used, the results were in concordance with the predicted values from CCD. As suspected, 0.1% supported greater laccase titer than 0.2% NH_4_Cl. Regardless of the NH_4_Cl concentration, a linear relationship between laccase titer and surface to volume ratio was observed, emphasizing the importance of surface to volume ratio for the production of metabolites and enzymes by fungi.

## 3. Experimental Section

### 3.1. Microorganism and culture conditions

A marine-derived basidiomycete *Cerrena unicolor* MTCC 5159 was used as the laccase source. It was maintained on Boyd & Kohlmeyer agar [34] prepared in half-strength sea water. The seed culture was developed by inoculating pieces of fungus in a low nitrogen mineral liquid medium [35]. This was kept under stationary conditions for 7 days at 30 °C. The mycelium was homogenized in Omni Macro-homogenizer (Model No. 17505, Marietta, GA, USA) for 5 s and this suspension was used as the inoculum. One mL inoculum (5% v/v) was transferred to Erlenmeyer flasks (250 mL) containing 20 mL of the culture medium and incubated at 30 °C under static conditions.

### 3.2. Culture medium

The culture medium contained a mineral salts basal medium consisting of 2 mL trace elements solution, 1 mL basal salts solution and 1 mL of 0.1% thiamine. The pH was adjusted to 4.5 using citrate-phosphate buffer and the final volume was made to 20 mL using distilled water. To this basal medium, glucose, NH_4_Cl, CuSO_4_, Tween 80 and NaCl were added at varying concentrations as per the requirement of the experiment. Trace elements solution contained: 0.5 g MnSO_4_, 0.08 g CdCl_2_, 0.1 g FeSO_4_.7 H_2_O, 0.1 g ZnSO_4_.7 H_2_O, 0.01 g AlK(SO_4_)_2_.12 H_2_O, 0.01 g H_3_BO_3_, 0.01 g Na_2_ MoO_4_.2 H_2_O dissolved in 1 L distilled water containing 1.5 g nitrilic triacetate, whose pH was adjusted to 6.5 with 1N KOH. This solution was filtered and stored in the dark at 4 °C. Basal salts solution contained 40 g KH_2_PO_4_, 10 g MgSO_4_ and 2 g CaCl_2_ in a total volume of 1,000 mL distilled water.

### 3.3. Screening of significant components using Plackett Burman Design

Screening of medium components was carried out using the PBD to determine their effect on biomass and laccase production. The five components used included glucose (carbon), NH_4_Cl (nitrogen), CuSO_4_ (inducer), Tween 80 (surfactant) and NaCl (salt). Low and high settings of each of these components were used to prepare a combination of eight Trials of the culture medium (Table 1). Inoculation was carried out using 5% (v/v) inoculum. An additional set of experiments was carried out with a Cu spike (0.375 mM) on day 6 of growth. Both sets of experiments were monitored every 3 days from the day of inoculation until day 15.

### 3.4. Effect of carbon and nitrogen interaction using Central Composite Design

Glucose and NH_4_Cl were used as the carbon and nitrogen source respectively. The concentrations of glucose and NH_4_Cl corresponding to the various coded levels and the experimental design are given in Table 5. Each Trial contained different combinations of glucose and NH_4_Cl. Copper sulphate at 0.375 mM, was added on day 6. Tween 80 was excluded from this medium. Laccase and biomass production were monitored on day 12.

### 3.5. Impact of addition of CuSO_4_ and Tween 80

The culture medium with 3.75% glucose, 0.25% NH_4_Cl and 0.1% NaCl was employed. To this, 0.375 mM CuSO_4_ was added along with varying concentrations (0% – 0.5%) of Tween 80 on day 6. Laccase and biomass production were monitored on day 12.

### 3.6. Effect of flask capacity

The culture medium contained 3% glucose, 0.1% NaCl and 0.1 or 0.2% NH_4_Cl. The flask sizes ranged from 100 mL to 500 mL capacity. The volume of the culture medium was maintained at 1/12.5 times the flask capacity. On day 6, 0.375 mM CuSO_4_ was added along with 0.25% Tween 80. Laccase and biomass production were measured on day 12.

### 3.7. Experimental design and statistical analysis

Plackett Burman Design was used to determine the significance of each of the medium components on biomass and laccase production. Table 1 represents the low and high settings for the five medium components under testing. A linear approach is considered sufficient for screening for the effects of these components:

(3) $$\text{Y}\;=\;\beta_{0}\;+\;\sum \beta_{i}X_{i}(i\;=\;1,\ldots \ldots .,\;\text{k})$$

Where Y is the estimated biomass or laccase production and *β_i_* is the regression coefficient.

The CCD was used to study the interactions between glucose (carbon) and NH_4_Cl (nitrogen). Table 5 represents the coded and uncoded variables for the two experimental variables. The design of the experiment along with the predicted and experimental values for laccase and biomass production is given in Table 5. For both, the screening and interaction studies, various measurements for each experimental set were carried out in triplicates. Each experimental set was carried out in duplicate. The yields reported are the mean of the duplicates. The relationship of the independent variables (A & B) and their response estimated (Y) as biomass or laccase production, were calculated by the second order polynomial equation:

(4) $$\text{Y}\;=\;\beta_{0}\;+\;(\beta_{1}*\text{A})\;+\;(\beta_{2}*\text{B})\;+\;(\beta_{11}*{\text{A}}^{2})\;+\;(\beta_{22}*{\text{B}}^{2})\;+\;(\beta_{12}*\text{A}*\text{B})$$

Where β_0_ is the constant coefficient, β_1_ and β_2_ are the linear coefficients; β_11_ and β_22_ are the quadratic coefficients whereas β_12_ is second order interaction coefficient. The statistical analysis using multiple regressions and MANOVA were performed with Statistica v 6.0 (StatSoft, Tulsa, Okla) software package.

### 3.8. Responses measured

Fungal biomass was estimated as dry weight. The mycelium was rinsed with distilled water and filtered through pre-weighed Whatman No.1 filter paper. This was dried in an oven until a constant weight was achieved. The difference in weight was considered as the mycelial dry weight and expressed as gram fungal dry weight per liter of medium (g L^−1^).

Laccase activity was estimated by measuring the rate of oxidation of ABTS spectrophotometrically using UV-Vis 2450 spectrophotometer (Shimadzu, Japan) [36]. Laccase present in the culture filtrate reacted with 1 mM ABTS prepared in glycine-HCl buffer (0.2 M; pH 3) to produce a green colored compound. The rate of formation of this product was measured spectrophotometrically at 405 nm at 60 °C, since the optimum pH and temperature for laccase assay were found to be 3 and 60 °C respectively [4]. Laccase activity was expressed in units, which is defined as 1 μmol of product formed per min per liter of culture filtrate (U L^−1^). Protein concentration was determined using the Bradford method [37] at 30 °C and expressed as gram protein per liter of culture filtrate (g L^−1^).

## 4. Conclusions

The temporal variation in laccase titer could be decreased by maintaining a constant acidic pH. Low concentrations of inorganic nitrogen and high concentrations of easily assimilable carbon favorably supported laccase production. A single addition of CuSO_4_ had a positive impact on laccase production whereas a repeat addition of CuSO_4_ on day 6 negatively impacted laccase production. Thus, a one time addition of CuSO_4_ on day 6 and response measurement on day 12 was found to be ideal. Seawater rather than NaCl favored biomass and laccase production in this marine-derived fungus. It was observed that a higher surface to volume ratio was required for better biomass production and this in turn positively affected laccase production. This requirement could be offset to a certain degree by the inclusion of a surfactant such as Tween 80.

## Figures and Tables

**Table 1.** Distribution of the low and high settings for each of the five variables with the coded and uncoded values in eight experimental Trials of the Plackett Burman Design.

**Trial**	**Variables**									
	**Coded**					**Uncoded**				
	**Glucose**	**NH_4_Cl**	**CuSO_4_**	**Tween 80**	**NaCl**	**Glucose (%)**	**NH_4_Cl (%)**	**CuSO_4_ (mM)**	**Tween 80 (%)**	**NaCl (%)**
**A**	−	−	−	−	+	0.9	0.2044	0.0375	0.05	1
**B**	−	−	+	+	−	0.9	0.2044	0.375	0.25	0.1
**C**	−	+	−	+	−	0.9	1.022	0.0375	0.25	0.1
**D**	−	+	+	−	+	0.9	1.022	0.375	0.05	1
**E**	+	−	−	+	+	4.5	0.2044	0.0375	0.25	1
**F**	+	−	+	−	−	4.5	0.2044	0.375	0.05	0.1
**G**	+	+	−	−	−	4.5	1.022	0.0375	0.05	0.1
**H**	+	+	+	+	+	4.5	1.022	0.375	0.25	1

The higher concentration of the variable is represented by + and the lower concentration by −.

**Table 2.** Laccase and biomass production based on the eight Trials (A to H) of both the spiked and unspiked Plackett Burman Design sets at different sampling intervals.

**Set**		**Biomass (g L^−1^)**					**Laccase (U L^−1^)**				

	**Trial**	**Sampling Interval (Days)**									
		**3**	**6**	**9**	**12**	**15**	**3**	**6**	**9**	**12**	**15**
**Unspiked**	**A**	0.96	1.21	1.30	1.08	1.70	807	901	888	48	3
	**B**	0.48	2.59	3.37	3.36	2.44	17	2926	2358	1393	760
	**C**	0.57	1.00	1.25	1.32	1.55	809	1651	792	3	220
	**D**	0.38	0.51	0.62	0.34	1.15	8	33	908	3	84
	**E**	0.89	1.85	2.60	2.52	2.12	6	1122	805	1070	156
	**F**	1.00	2.52	3.68	3.47	2.56	10	1815	1744	1787	662
	**G**	1.08	0.93	1.61	1.49	1.80	802	541	178	178	122
	**H**	0.30	1.17	1.79	1.75	1.72	3	253	825	624	434

**Spiked**	**A’**	0.96	1.21	0.93	1.21	1.50	807	901	502	254	145
	**B’**	0.48	2.59	3.62	3.12	2.41	17	2926	320	74	67
	**C’**	0.57	1.00	1.08	1.10	1.24	809	1651	288	82	72
	**D’**	0.38	0.51	0.11	0.36	0.76	8	33	301	88	73
	**E’**	0.89	1.85	2.44	2.26	2.09	6	1122	302	62	65
	**F’**	1.00	2.52	3.64	3.51	2.62	10	1815	110	91	71
	**G’**	1.08	0.93	1.15	1.62	1.55	802	541	100	102	82
	**H’**	0.30	1.17	1.58	1.43	1.37	3	253	67	54	45

Medium composition of Trials A to H is explained in Table 1. Trials A to H are collectively referred to as the ‘unspiked’ set where CuSO_4_ was added only at the time of inoculation. Trials A’ to H’ are collectively referred to as the ‘spiked’ set and are identical to the corresponding Trials A to H with the exception that an additional 0.375 mM of CuSO_4_ was added to each of these Trials on day 6. The effect of this CuSO_4_ spike was recorded from day 9 onwards, hence are illustrated in blue.

**Table 3.** Degree of positive and negative effects of medium components on biomass production according to the Plackett Burman Design.

**Variables**	**Sampling Interval (Days)**														
	**3**			**6**			**9**			**12**			**15**		

	**B**	**Std. Err. of B**	**p-level**	**B**	**Std. Err. of B**	**p-level**	**B**	**Std. Err. of B**	**p-level**	**B**	**Std. Err. of B**	**p-level**	**B**	**Std. Err. of B**	**p-level**

***Intercept***	*1.250*	*0.016*	*0.0000*	*1.916*	*0.016*	*0.0000*	*2.310*	*0.060*	*0.0000*	*2.107*	*0.092*	*0.0000*	*2.142*	*0.152*	*0.0000*
**Glucose**	0.061	0.003	0.0000	0.081	0.003	0.0000	0.217	0.010	0.0000	0.217	0.016	0.0000	0.095	0.026	0.0043
**NH_4_Cl**	−0.305	0.012	0.0000	−1.393	0.012	0.0000	−1.739	0.045	0.0000	−1.692	0.069	0.0000	−0.794	0.113	0.0000
**CuSO_4_**	−0.994	0.029	0.0000	1.327	0.030	0.0000	1.999	0.108	0.0000	1.854	0.167	0.0000	0.525	0.275	0.0849
**Tween 80**	−1.469	0.048	0.0000	1.798	0.050	0.0000	2.242	0.182	0.0000	3.213	0.282	0.0000	0.780	0.464	0.1235
**NaCl**	−0.172	0.011	0.0000	−0.639	0.011	0.0000	−1.002	0.041	0.0000	−1.096	0.063	0.0000	−0.465	0.103	0.0011
	Adjusted r^2^ = 0.9958			Adjusted r^2^ = 0.999			Adjusted r^2^ = 0.9952			Adjusted r^2^ = 0.9890			Adjusted r^2^ = 0.84900		
	S.E.E.: 0.0193			S.E.E.: 0.020			S.E.E.: 0.0729			S.E.E.: 0.1129			S.E.E.: 0.18545		

	**Sampling Interval (Days)**														
**Variables**	A spike of 0.375 mM CuSO_4_ was troduced on day 6.						**9**			**12**			**15**		

							**B**	**Std. Err. of B**	**p-level**	**B**	**Std. Err. of B**	**p-level**	**B**	**Std. Err. of B**	**p-level**
	
***Intercept***							*2.126*	*0.092*	*0.0000*	*2.364*	*0.160*	*0.0000*	*2.141*	*0.1604*	*0.0000*
**Glucose**							0.213	0.016	0.0000	0.211	0.027	0.0000	0.119	0.0272	0.0014
**NH_4_Cl**							−2.053	0.069	0.0000	−1.710	0.120	0.0000	−1.130	0.1197	0.0000
**CuSO_4_**							2.481	0.167	0.0000	1.653	0.290	0.0002	0.578	0.2900	0.0741
**Tween 80**							3.605	0.282	0.0000	1.502	0.489	0.0118	0.840	0.4894	0.1170
**NaCl**							−1.231	0.063	0.0000	−1.137	0.109	0.0000	−0.583	0.1088	0.0003
							Adjusted r^2^ = 0.9919			Adjusted r^2^ = 0.96483			Adjusted r^2^ = 0.90251		
							S.E.E.: 0.1129			S.E.E.: 0.19546			S.E.E.: 0.19576		

Main effects (B) *i.e.*, biomass production (g L^−1^) and standard error of B for uncoded values. p-level is equivalent to 0.05%. S.E.E = Standard Error of Estimate.

**Table 4.** Degree of positive and negative effects of medium components on laccase production according to the Plackett Burman Design.

**Variables**	**Sampling Interval (Days)**														
	**3**			**6**			**9**			**12**			**15**		

	**B**	**Std. Err. of B**	**p-level**	**B**	**Std. Err. of B**	**p-level**	**B**	**Std. Err. of B**	**p-level**	**B**	**Std. Err. of B**	**p-level**	**B**	**Std. Err. of B**	**p-level**
	
***Intercept***	952	146	0.000	2375	30	0.000	1471	26	0.000	540	86	0.000	198	37	*0.000*
**Glucose**	−57	25	0.044	−124	5	0.000	−97	4	0.000	154	15	0.000	21	6	0.007
**NH_4_Cl**	239	109	0.053	−1310	23	0.000	−946	19	0.000	−1067	64	0.000	−221	28	0.000
**CuSO_4_**	−1768	264	0.000	602	55	0.000	2350	46	0.000	1858	155	0.000	1067	67	0.000
**Tween 80**	−991	446	0.051	3329	92	0.000	1329	78	0.000	1343	262	0.000	873	113	0.000
**NaCl**	−226	99	0.046	−1284	20	0.000	−457	17	0.000	−449	58	0.000	−302	25	0.000
	Adjusted r^2^ = 0.7997			Adjusted r^2^ = 0.998			Adjusted r^2^ = 0.998			Adjusted r^2^ = 0.9761			Adjusted r^2^ = 0.9725		
	S.E.E.: 178.5200			S.E.E.: 36.884			S.E.E.: 31.245			S.E.E.: 104.7401			S.E.E.: 45.1227		

**Variables**	**Sampling Interval (Days)**														
	A spike of 0.375 mM CuSO_4_ was introduced on day 6.						**9**			**12**			**15**		
	
							**B**	**Std. Err. of B**	**p-level**	**B**	**Std. Err. of B**	**p-level**	**B**	**Std. Err. of B**	**p-level**
	
***Intercept***							507	9.5	0.000	228	29	0.000	143	10	0.000
**Glucose**							−58	1.6	0.000	−13	5	0.024	−7	2	0.003
**NH_4_Cl**							−146	7.1	0.000	−47	22	0.054	−23	7	0.010
**CuSO_4_**							−292	17.1	0.000	−143	53	0.022	−80	18	0.001
**Tween 80**							−45	28.9	0.151	−329	89	0.004	−153	30	0.000
**NaCl**							98	6.4	0.000	30	20	0.155	10	7	0.161
							Adjusted r^2^ = 0.9956			Adjusted r^2^ = 0.66959			Adjusted r^2^ = 0.82375		
							S.E.E.: 11.5611			S.E.E.: 35.46043			S.E.E.: 11.88795		

Main effects (B) i.e laccase production (U L^−1^); standard error of B for uncoded values. p-level is equivalent to 0.05%. S.E.E = Standard Error of Estimate.

**Table 5.** Central Composite Design matrix with coded and uncoded values chosen for glucose and NH_4_Cl with the experimental, predicted and residual values for biomass and laccase production.

**Trial**	**Variables**				**Values**					

	**Coded**		**Uncoded**		**Experimental**		**Predicted**		**Residual**	

	**Glucose**	**NH_4_Cl**	**Glucose (%)**	**NH_4_Cl (%)**	**Biomass (g L^−1^)**	**Laccase (U L^−1^)**	**Biomass (g L^−1^)**	**Laccase (U L^−1^)**	**Biomass (g L^−1^)**	**Laccase (U L^−1^)**

**1**	−1	−1	1.25	0.25	2.0	374	2.01	271	−0.01	103
**2**	−1	+1	1.25	0.75	1.56	254	1.80	372	−0.24	−118
**3**	+1	−1	3.75	0.25	2.62	917	2.38	690	0.24	227
**4**	+1	+1	3.75	0.75	2.24	135	1.81	−2	0.43	137
**5**	0	0	2.5	0.5	1.93	282	2.09	233	−0.16	49
**6**	− α	0	0.7	0.5	1.87	156	1.76	249	0.11	−93
**7**	+ α	0	4.3	0.5	2.43	153	2.03	281	0.4	−128
**8**	0	− α	2.5	0.14	2.26	416	2.38	621	−0.12	−205
**9**	0	+ α	2.5	0.86	1.79	325	1.82	196	−0.03	129
**10**	0	0	2.5	0.5	2.0	271	2.09	233	−0.09	38
**11**	0	0	2.5	0.5	1.9	286	2.09	233	−0.19	53
**12**	0	0	2.5	0.5	1.89	243	2.09	233	−0.2	10

## Abbreviations:

ABTS: [2,2′-azino-bis-(3-ethylbenzothazoline-6-sulfonate)];
CCD: Central Composite Design;
PBD: Plackett Burman Design
